# Is Abdominal Aortic Aneurysm Behavior after Endovascular Repair Associated with Aneurysm Wall Density on Computed Tomography Angiography?

**DOI:** 10.3390/medicina55080406

**Published:** 2019-07-25

**Authors:** Arminas Skrebūnas, Givi Lengvenis, Inga Urtė Builytė, Rūta Žulpaitė, Rytis Bliūdžius, Petras Purlys, Tomas Baltrūnas, Nerijus Misonis, Mindaugas Matačiūnas, Germanas Marinskis, Donatas Vajauskas

**Affiliations:** 1 Clinic of Cardiovascular Diseases, Faculty of Medicine, Vilnius University, 01513 Vilnius, Lithuania; 2 Centre of Cardiology and Angiology, Vilnius University Hospital Santaros Klinikos, 08410 Vilnius, Lithuania; 3 Centre of Radiology and Nuclear Medicine, Vilnius University Hospital Santaros Klinikos, 08410 Vilnius, Lithuania; 4 Department of Radiology, Medical Academy, Lithuanian University of Health Sciences, 44307 Kaunas, Lithuania

**Keywords:** abdominal aortic aneurysm, EVAR, follow-up, AAA volume, growing AAA, wall density

## Abstract

*Background and objectives:* Abdominal aortic aneurysm (AAA) growth is unpredictable after the endovascular aneurysm repair (EVAR). Continuing aortic wall degradation and weakening due to hypoxia may have a role in post-EVAR aneurysm sac growth. We aimed to assess the association of aortic wall density on computed tomography angiography (CTA) with aneurysm growth following EVAR. *Materials and Methods:* A total of 78 patients were included in the study. The control group consisted of 39 randomly assigned patients without aortic pathology. Post-EVAR aneurysm sac volumes on CTA were measured twice during the follow-up period to estimate aneurysm sac behavior. A maximum AAA sac diameter, aortic wall and lumen densities in Hounsfield units (HU) on CTA were measured. A relative aortic wall density (the ratio of aortic wall to lumen densities) was calculated. A statistical data analysis was performed using standard methods. *Results:* An increase in the AAA sac volume was observed in 12 (30.8%) cases. Median relative aortic wall density on CTA scores in both the patient and the control group at the level of the diaphragm were similar: 0.15 (interquartile range (IQR), 0.11–0.18) and 0.16 (IQR 0.11–0.18), *p* = 0.5378, respectively. The median (IQR) relative aortic wall density score at the level of the maximum AAA diameter in the patient group was lower than at the level below renal arteries in the control group: 0.10 (0.07–0.12) and 0.17 (0.12–0.23), *p* < 0.0001, respectively. The median (IQR) relative growing AAA sac wall density score was lower than a relative stable/shrinking AAA sac wall density score: 0.09 (0.06–0.10) and 0.11 (0.09–0.13), *p* = 0.0096, respectively. *Conclusions:* A lower aortic aneurysm wall density on CTA may be associated with AAA growth after EVAR.

## 1. Introduction

The development of the abdominal aortic aneurysm (AAA) is a complex process in which hypoxia [1,2], inflammation [3,4,5] and biomechanical wall stress [6,7] are considered key pathological factors. These processes lead to extracellular matrix degradation and remodeling in the aortic media, loss of structural integrity and consequential dilation of aortic wall [3,8].

Endovascular aneurysm repair (EVAR) nowadays is frequently chosen for the prevention of further AAA growth and rupture. In comparison with an open AAA repair, the endovascular approach has several advantages, such as a shorter hospital stay, lower perioperative mortality [9,10] and even a comparatively longer survival period [11]. Nevertheless, the rate of reinterventions has been found to be much more frequent for patients who underwent EVAR [12,13]. The endovascular approach does not eliminate the aneurysm sac and its exclusion is not always definite, therefore, the risk of further aneurysm growth and rupture remains, mainly caused by endoleaks or stent-graft migration [9,10,14,15]. Factors that prevent the aneurysm sac from shrinking or driving its further expansion after the endovascular procedure are still not completely understood, especially when it occurs due to endotension (without visible endoleak). It has been suggested that biologically active intraluminal thrombus (ILT), which contains a variety of inflammatory factors as well as proteolytic enzymes, may have a role in post-EVAR aneurysm sac growth due to wall degradation and weakening [16,17]/ However, the results of previous studies differ considerably [18,19,20,21]. While aneurysm sac behavior still remains unpredictable after EVAR, constant follow-up imaging, including computed tomography angiography (CTA), is necessary [22]. Several predictive models for complications after the endovascular AAA treatment have been proposed, which would allow for customized surveillance, especially considering the frequency of CTAs, according to individual patient’s risk [23,24].

A growing body of evidence shows that the aneurysmatic aortic wall is injured under hypoxic conditions, which leads to compensatory neovascularization, a higher burden of reactive oxygen species (ROS) and inflammation and destruction of the normal histological structure [1,2]. An increased aortic wall density on CTA or contrast-enhanced magnetic resonance imaging (MRI) is associated with hypervascularization, an inflammation or atherosclerosis within the aortic wall [25]. A decreased aneurysm wall density may correspond with ischemic lesions within it. We hypothesize that pathological changes of the blood supply to the aortic wall may also have an impact on the AAA sac behavior after EVAR. Therefore, we aimed to assess the association of aortic wall density on CTA with the aneurysm sac growth following endovascular repair.

## 2. Materials and Methods

The study was approved by the Vilnius Regional Biomedical Research Ethics Committee (registered 13/12/2016 reg. 158200-16-877-386) and conducted according to the principles of the Helsinki Declaration. This is a longitudinal observational cohort study, which was conducted in a university hospital between January 2007 and September 2017.

A study cohort included patient and control groups. The former group consisted of patients undergoing an elective EVAR. A total of 107 patients underwent EVAR during the period. The inclusion criteria were as follows: written informed consent, age over 50 years, at least two CTA scans after EVAR during the first 2 years of follow-up and no visible endoleak. A total of 39 patients met the inclusion criteria. A clinical control group—which was necessary to compare the density of the aortic wall at corresponding levels of the normal and aneurysmatic aorta above and below the lesion of patients over 50 years old, for whom CTA scans of chest and abdomen were performed and no aortic pathology detected—was also included in the study. A total of 1847 patients underwent CTA scans during the period. A total of 39 patients were selected using simple random sampling. A total of 78 patients were further analyzed in the study, their baseline data were collected from medical documents.

A total of 117 scans in the patient group were evaluated by two independent radiologists. The threshold for clinically relevant diametric AAA sac expansion after EVAR remains unclear for patients without endoleaks. It was decided to measure the aortic aneurysm volume instead of the diameter as a more accurate method [26] because up to a 2 mm error in the measuring diameter may occur using a non-electrocardiographic (ECG)-gated CTA [27]. The aneurysm sac volumes were measured twice during the follow-up period—on the first and the last CTA scan after EVAR—and changes between the measurements were calculated. The means of two radiologists’ AAA volume measurements were used for further analysis. The data of the maximum aortic aneurysm sac diameter on the CTA scan were collected.

All the CTA scans were performed using helical CT scanners GE (General Electric Healthcare, Waukesha, Wisconsin, United States) LightSpeed VCT (until 2012) and GE Discovery CT750 HD (since 2013) under a set acquisition protocol. Contrast-enhanced images were obtained after injecting 70–120 mL of non-ionic intravenous contrast matter. Images were initially reconstructed in axial planes with a slice thickness of 0.625 mm.

CTA images were transferred to Vitrea (Vital Images, Inc., Minnetonka, Minnesota, United States) picture archiving and communication system (PACS) archiving for the initial assessment of the aneurysm sac and stent graft location that was performed using the Vital Vitrea v.6.7.2 software. For measurements of aneurysm volume and lumen Vitrea Advanced aorta, the “stent graft planning” protocol was used. An automated 3D segmentation by the software was performed, followed by manual adjustments to the centerline and outlines of the aneurysm and lumen in each slice where it was considered inaccurate. On average, the processing and evaluation of a single examination took 26.5 ± 2.3 min and 29.1 ± 1.0 min for two radiologists accordingly. Volumetric measurements included a portion of the abdominal aorta, the aneurysm and the iliac arteries covered by stentgraft. The software computed the volume of the aneurysm (including the wall of the aorta) in cubic centimeters (mL).

**Figure 1:**
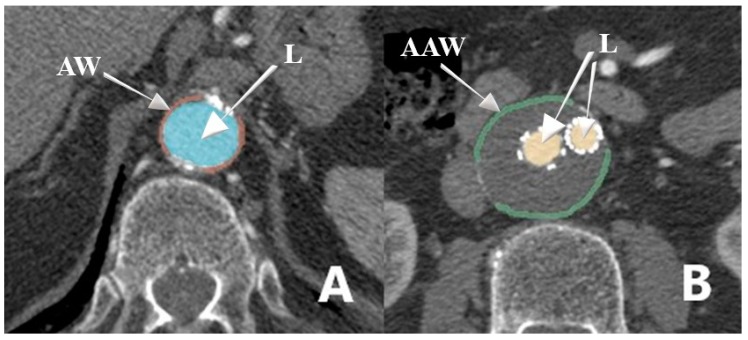
The segmentation of the abdominal aorta at the level of the diaphragm (**A**) and at the level of the maximum abdominal aortic aneurism (AAA) diameter in the patient group (**B**). AW: aortic wall; AAW: aortic aneurysm wall; L: lumen.

Manual segmentation of the abdominal aortic wall and lumen from the CTA scan was performed at two different levels: at the level of the diaphragm and at the level of the maximum AAA diameter in the patient group or at the level below the renal arteries in the control group (Figure 1). Aortic wall segments free of plaques and artifacts were selected. Densities in Hounsfield units (HU) of the aortic wall and lumen were measured. The means of the two radiologists’ density measurements were used for further analysis. Because of the patients’ circulation abnormalities, an amount of injected intravenous contrast material and the delay of the CTA scan, the aortic wall and lumen density may vary. Considering this, the ratio of aortic or aortic aneurysm wall density to lumen density was calculated (relative aortic wall density).

Data entry, calculations and statistical analysis were carried out using Microsoft Office Excel 2016.

Descriptive statistics of patient baseline characteristics and the interval between 2 CTA scans were calculated. The Bland–Altman analysis was performed to determine the correlation between measurements done by two independent radiologists. The bias was calculated as the average difference between their results. Parametric data were presented in terms of the mean value and standard deviation (SD). All the ordinal data were presented as an absolute number and percentage prevalence in the study population. Median scores of the relative aortic wall density in groups and subgroups were compared using the Mann–Whitney U test. Interquartile range and median values were calculated to provide descriptive statistics for non-parametric tests. All the reported *p*-values are two-sided, and a *p*-value of 0.05 was considered the threshold of statistical significance.

## 3. Results

### 3.1. Baseline Characteristics

There were 107 patients included in the study group initially. A total of 14 (13.1%) patients were excluded from the analysis because endoleaks had developed during the surveillance period. Ten (9.3%) patients who had experienced other non-endoleak related complications such as limb occlusion, stent graft migration after EVAR, were also excluded. A total of 44 (41.1%) patients were excluded from further analysis because of incomplete or no surveillance. Overall, 39 (36.5%) patients were studied. There were 4 (10.3%) women; 35 (89.7%) men; the median (IQR) age was 71 (63–76) years.

The control group consisted of 39 patients without aortic pathology detected on CTA. There were 6 (15.4%) women; 33 (84.6%) men; the median (IQR) age was 73 (64–78) years.

There was no difference between the groups regarding the gender, age, cardiovascular risk factors and chronic obstructive pulmonary disease (COPD) (Table 1).

### 3.2. Reproducibility

**Figure 2:**
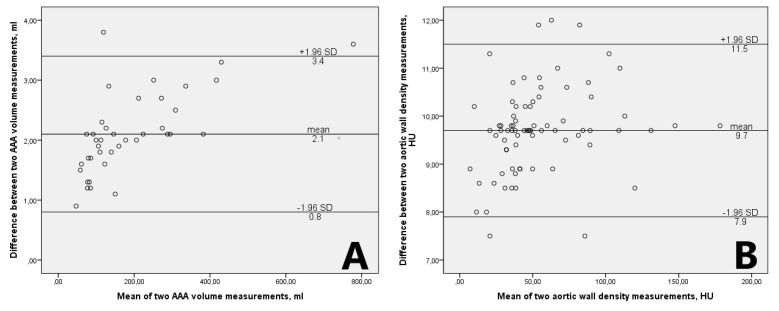
The Bland–Altman plots of abdominal aortic aneurysm volume measurements (**A**) and aortic wall density measurements (**B**). AAA: abdominal aortic aneurysm; HU: Hounsfield units; SD: Standard deviation.

The Bland–Altman plot was used to assess interobserver variability, finding no significant difference between the median of aortic aneurysm volume measurements (*p >* 0.05), the mean difference being 2.1 ± 0.67 mL, and aortic wall density measurements (*p* > 0.05), the mean difference being 9.7 ± 0.91 HU (Figure 2A,B).

### 3.3. Volumetric AAA Sac Changes

The median (IQR) follow-up time was 679 (427–737) days. The CT scans were not performed at the same time intervals for each patient as a follow-up algorithm was not developed at the time. The first follow-up CT was performed after a median (IQR) of 37 (31–91) days and the last one—after a median (IQR) of 679 (427–737) days after EVAR.

An increase in AAA sac volume was observed in 12 (30.8%) cases. The mean AAA volume increase in percentage was 13.3 ± 8.1%. For the remaining 27 (69.2%) cases, no increase in aneurysm sac volume was observed. The average AAA volume decrease was 25.3 ± 14.8%. According to the postoperative volumetric AAA sac changes, all patients after EVAR were subdivided into 2 subgroups: growing AAA sac subgroup and stable or shrinking AAA sac subgroup.

There was no difference between the subgroups regarding the gender, age, cardiovascular risk factors and COPD (Table 2).

### 3.4. Aortic Wall Densities on CTA within the Control Group

An aortic wall density in HU was measured at two different levels: at the level of the diaphragm and at the level below the renal arteries for the same patient. A relative aortic wall density was calculated. Median (IQR) relative density scores were similar at the level of the diaphragm and below the renal arteries: 0.16 (0.11–0.18) and 0.17 (0.12–0.23), *p* = 0.3030, respectively.

### 3.5. Aortic and Aneurysm Wall Densities on CTA within the Patient Group

A median (IQR) relative aortic aneurysm wall density score was lower at the level of the maximum AAA diameter (0.10 (0.07–0.12)) than at the level of the diaphragm (0.15 (0.11–0.18)), *p* < 0.0001.

### 3.6. Differences between Aortic and Aneurysm Wall Densities on CTA between the Groups

The groups were similar in respect to the median (IQR) relative aortic wall density scores at the level of the diaphragm: 0.15 (0.11–0.18) in the patient group and 0.16 (0.11–0.18) in the control group, *p* = 0.5378. A median (IQR) relative aortic aneurysm wall density score at the level of the maximum AAA diameter in the patient group was lower than a median (IQR) relative aortic wall density score below the renal arteries in the control group: 0.10 (0.07–0.12) and 0.17 (0.12–0.23), *p* < 0.0001, respectively.

### 3.7. Differences between Aortic and Aneurysm Wall Densities on the CTA between the Subgroups

The growing AAA sac subgroup and the stable or shrinking AAA sac subgroup were similar in respect to the median (IQR) relative aortic wall density scores at the level of the diaphragm: 0.16 (0.11–0.20) and 0.14 (0.10–0.17), *p* = 0.1592, respectively. A median (IQR) relative aortic aneurysm wall density score at the level of maximum AAA diameter in the growing AAA sac subgroup was lower than in the stable or shrinking AAA sac subgroup: 0.09 (0.06–0.10) and 0.11 (0.09–0.13), *p* = 0.0096, respectively.

All the differences between aortic and aneurysm wall densities on the CTA between the groups and subgroups are shown in Table 3.

## 4. Discussion

One of the main findings of the study is that the aortic aneurysm wall density is lower compared to the normal aorta. Secondly, the wall density of the growing AAA after EVAR is lower in comparison to the stable or shrinking AAA. Lastly, the measurements of the aortic wall density on CTA, both in the patient and the control group at the level of the diaphragm and within the control group at different aortic levels are similar.

To the best of our knowledge, this is the first study that investigates the association between the AAA wall density on CTA and aneurysm growth after endovascular repair. The contrast material’s distribution into tissues is determined by the arterial blood supply. Higher aortic wall densities on CTA may show not only hypervascularization but also calcification, intramural hematoma and inflammation of the damaged aortic wall in the general population. Reduced amounts of contrast material on the CTA may reveal ischemic areas because of the perfusion defects within tissues. Varga-Szemes et al. showed CT myocardial perfusion imaging as being useful as an add-on to coronary CTA to increase the specificity for hemodynamically relevant ischemic myocardial lesions [28]. Our findings show the aortic aneurysm wall hypoperfusion which may support an ischemic AAA progression theory.

It is considered that hypoxic conditions in the aortic wall may be a key factor causing the development and progression of AAA. Normally, the aortic tissue is supplied from the luminal blood flow, as well as from the adventitial vasa vasorum (VV). An infrarenal abdominal aorta has a smaller net of VV in contrast to the thoracic aorta; therefore, it is particularly susceptible to hypoxia [29]. A thick intraluminal thrombus causes localized ischemia in the underlying aortic wall [1]. Researchers have found that ILT thickness is related to the increased vascular smooth muscle cells (VSMC) apoptosis, elastin degradation and higher levels of proteolytic enzyme matrix metalloproteinase (MMP)-2 in the AAA wall, which leads to the impairment of the normal histological structure and weakening of the aortic wall [19]. A recent study by Haller et al. showed that a thicker ILT is associated with aneurysm rupture at smaller diameters and lower wall stress values [30]. Arteriosclerotic degeneration and significant stenosis of adventitial VV have been recently found in histological specimens of human AAA [29,31,32]. Moreover, an experimental rodent model has been created, which demonstrates that the creation of hypoxic conditions in the aortic wall by the mechanical cessation of the blood flow through VV gradually causes the infrarenal aortic aneurysm to develop [29,32]. Hypoxia-inducible factor-1 (HIF-1), which is secreted by the human organism as an adaptive reaction against hypoxia, has been found significantly upregulated in human and in experimental AAA walls [29,31,32,33,34,35]. HIF-1 triggers an inflammatory response, the increased influx of macrophages [34] and the subsequent augmented production of MMPs, which are known to play an essential role in the destruction of the normal histological structure and the weakening of the AAA wall [36,37]. Additionally, chronic hypoxia impairs the oxidant/antioxidant balance, which leads to increased levels of ROS in the tissue [2]. This may explain our results—the structure of the ischemic aneurysm sac may be less stable and weaker; therefore, it is more likely to expand or fail to shrink after endovascular stentgraft implantation.

Our results are contrary to those of other authors who have emphasized the role of medial neoangiogenesis in the development and progress of the AAA disease. Medial neovascularization and overexpression of proangiogenic factors, such as vascular endothelial-derived growth factor (VEGF), have been detected in human AAAs [38,39,40] as well as in experimental aortic aneurysms of mice models [40]. It has been demonstrated that microvessel density, especially of immature ones, and the expression of proangiogenic factors in the medial layer, are significantly increased in the rupture edge of the ruptured AAAs in comparison to the anterior wall of the ruptured aneurysms and the wall of the non-ruptured AAAs [38]. Moreover, a number of experimental models have shown the effectiveness of antiangiogenic therapy in the suppression of AAA growth [41,42,43,44]. Inflammation is considered to be both a stimulus and a consequence of neoangiogenesis. Recent studies have proved that the extent of vascularization (the number of microvessels per high power field), as well as the expression of the vascular endothelial growth factor (VEGF), are significantly increased with the growing inflammatory activity in the AAA wall [39,45]. Exceptionally highly permeable immature vessels have been found in the center of inflammatory infiltrates, which show the active process of neovascularization [45]. Neovessels allow a higher influx of inflammatory cells into the aortic wall, which secretes proangiogenic factors such as VEGF-A, MMPs, C-C motif chemokine ligand (CCL) 2, CCL5, interleukin-8 and induce further angiogenesis [33,46]. Furthermore, not only inflammatory cells but also endothelial cells of neovessels themselves are a source of MMPs [47].

Hypoxia itself acts as a trigger for the compensatory neovascularization—the previously mentioned HIF-1 is a well-known proangiogenic mediator [33,34,35]. Other angiogenesis-stimulating factors, such as VEGF, nitric oxide synthase and cyclo-oxygenase-2 are also overexpressed under hypoxic conditions [42]. Wang et al. recently demonstrated that treatment with HIF-1a inhibitor suppresses the growth of experimental AAAs by the attenuation of mural angiogenesis, as well as the accumulation of inflammatory cells, medial elastin degradation and smooth muscle cell depletion [33]. In the study by Mäyränpää et al., thrombus-covered aneurysm specimens contained a significantly higher cluster of differentiation (CD) 31 messenger RNA (mRNA)—an immunohistochemical marker of endothelial cells—levels compared to specimens without thrombus [48]. However, no correlation between the CD31 vascularity and ILT thickness was found, which may indicate that the mechanism of AAA wall angiogenesis is complex [45].

The connection between the extent of medial neovascularization in the AAA wall and maximal aneurysm diameter is also controversial. Scott et al. showed a significant positive correlation between the AAA diameter and CD31 vascularity [45]. In another experimental study, VEGF receptor expression, as determined by a single-chain and labeled with Cy5.5 VEGF fluorescent imaging and VEGF receptor 2 immunostaining in the AAA wall, was also significantly higher with an increasing aneurysm diameter [41]. Conversely, a contrast-enhanced-MRI based study revealed that microvascular flow, permeability and surface area in the AAA walls, described as the transfer constant, only moderately correlates with the maximal diameter of the AAA [49].

Our results did not show a higher aortic wall density which may signify an increased vascularization in the growing aneurysm subgroup after EVAR, but, on the contrary, the perfusion of the aneurysm wall in that subgroup seemed to be lower. It is rather difficult to compare our study with previously mentioned works since most of them are based on histological but not radiological studies of the AAA wall. Moreover, none of them evaluated the changes of the aortic aneurysm wall perfusion after stentgraft implantation.

There are several limitations to our study. The most obvious limitation is a small sample size which may increase the likelihood of a type II error. The sample size was mostly reduced by the loss of patients at the follow-up due to underdeveloped surveillance strategies in the country. It is important to mention that few centers perform EVARs on a relatively small population, which may also affect the sample size. Despite the significant results of our study, further analysis is necessary for the selection of patients to whom this method may be useful in predicting the AAA behavior after EVAR as the measurement of the aortic aneurysm wall density is a time-consuming method. We did not evaluate the aortic aneurysm wall density on CTA before EVAR, which could be important in suspecting the growth of an aneurysm following endovascular repair. We did not have data on the aortic aneurysm wall density changes outside the study period. It may be crucial to know if the endovascular repair itself or other factors lead to a decreased perfusion of the aortic aneurysm wall.

## 5. Conclusions

This study demonstrated an association between the lower AAA wall density on CTA and the greater aneurysm sac growth after EVAR. This possibly corresponds to the aneurysm wall hypoperfusion and ischemia, which may impact the aneurysm progression. An AAA wall density measurement on CTA may be a valuable radiological indicator for predicting sac growth after endovascular repair. However, further studies are instrumental.

## Figures and Tables

**Table 1:** The baseline characteristics of the patients.

Characteristic	Patient Group	Control Group	*p*-Value
Age (median (IQR), years	71 (63–76)	73 (64–78)	0.215
Gender			
Male	35 (89.7%)	31 (79.5%)	0.347
Female	4 (10.3%)	8 (20.5%)	
Cardiovascular risk factors			
Smoker	9 (23.1%)	10 (25.6%)	0.968
Hypertension	34 (87.2%)	31 (79.5%)	0.309
Diabetes	4 (10.3%)	8 (20.5%)	0.334
COPD	6 (15.4%)	5 (12.8%)	0.368

IQR: interquartile range; COPD: chronic obstructive pulmonary disease; statistical significance: *p* < 0.05.

**Table 2:** The characteristics of the patients in the subgroups.

Characteristic	Growing AAA Sac Subgroup	Stable/Shrinking AAA Sac Subgroup	*p*-Value
Age (median (IQR), years	72 (63–80)	70 (62–74)	0.552
Gender			
Male	12 (100%)	23 (85.2%)	0.292
Female	0 (0%)	4 (14.8%)	
Cardiovascular risk factors			
Smoker	2 (16.7%)	7 (25.9%)	0.681
Hypertension	11 (91.7%)	23 (85.2%)	0.540
Diabetes	1 (8.3%)	3 (11.1%)	0.439
COPD	1 (8.3%)	5 (18.5%)	0.646

AAA: abdominal aortic aneurysm; IQR: interquartile range; COPD: chronic obstructive pulmonary disease; *p* < 0.05.

**Table 3:** The differences between the aortic and aneurysm wall densities on the computed tomography angiography (CTA).

Relative Aortic and Aneurysm Wall Densities	Patient Group		Control Group	*p*-Value
	Growing AAA Sac Subgroup	Stable/Shrinking AAA Sac Subgroup		
at the level of diaphragm	0.15 (IQR, 0.11–0.18)		0.16 (IQR, 0.11–0.18)	0.5378
	0.16 (IQR, 0.11–0.20)	0.14 (IQR, 0.10–0.17)		0.1592
at the level of maximum AAA diameter/below the renal arteries	0.10 (IQR, 0.07–0.12)		0.17 (IQR, 0.12–0.23)	<0.0001
	0.09 (IQR, 0.06–0.10)	0.11 (IQR, 0.09–0.13)		0.0096

AAA: abdominal aortic aneurysm; IQR: interquartile range; *p* < 0.05.
